# Terahertz reflectometry imaging for low and high grade gliomas

**DOI:** 10.1038/srep36040

**Published:** 2016-10-26

**Authors:** Young Bin Ji, Seung Jae Oh, Seok-Gu Kang, Jung Heo, Sang-Hoon Kim, Yuna Choi, Seungri Song, Hye Young Son, Se Hoon Kim, Ji Hyun Lee, Seung Joo Haam, Yong Min Huh, Jong Hee Chang, Chulmin Joo, Jin-Suck Suh

**Affiliations:** 1 YUHS-KRIBB Medical Convergence Research Institute, Yonsei University College of Medicine, Seoul, Republic of Korea; 2 Department of Neurosurgery, Brain Tumor Center, Brain Research Institute, Severance Hospital, Yonsei University College of Medicine, Seoul, Republic of Korea; 3 Department of Mechanical Engineering, Yonsei University, Seoul, Republic of Korea; 4 Applied Electromagnetic Wave Research Center, Korea Electrotechnology Research Institute, Ansan, Republic of Korea; 5 Severance Biomedical Science Institute, Severance Hospital, Yonsei University College of Medicine, Seoul, Republic of Korea; 6 Department of Pathology, Severance Hospital, Yonsei University College of Medicine, Seoul, Republic of Korea; 7 Department of Chemical and Biomolecular Engineering, Yonsei University, Seoul, Republic of Korea; 8 Department of Radiology, Severance Hospital, Yonsei University College of Medicine, Seoul, Republic of Korea

## Abstract

Gross total resection (GTR) of glioma is critical for improving the survival rate of glioma patients. One of the greatest challenges for achieving GTR is the difficulty in discriminating low grade tumor or peritumor regions that have an intact blood brain barrier (BBB) from normal brain tissues and delineating glioma margins during surgery. Here we present a highly sensitive, label-free terahertz reflectometry imaging (TRI) that overcomes current key limitations for intraoperative detection of World Health Organization (WHO) grade II (low grade), and grade III and IV (high grade) gliomas. We demonstrate that TRI provides tumor discrimination and delineation of tumor margins in brain tissues with high sensitivity on the basis of Hematoxylin and eosin (H&E) stained image. TRI may help neurosurgeons to remove gliomas completely by providing visualization of tumor margins in WHO grade II, III, and IV gliomas without contrast agents, and hence, improve patient outcomes.

Around 28% of all primary brain tumors and 80% of primary malignant brain tumors are gliomas^1^. Many malignant glioma types, especially glioblastoma (GBM), have a poor prognosis even though aggressive therapy including surgery, radiotherapy, and chemotherapy are performed^2^. Several prognostic factors for better outcomes in glioma patients have been reported, which include age, genetic mutations, achievement of gross total resection (GTR)^3^,^4^,^5^,^6^. Among these factors, achievement of GTR was found to be crucial for the better prognosis^7^,^8^,^9^,^10^. Incomplete resection, even in low grade gliomas, leads to higher probability of recurrence and shorter survival by residual tumor^11^,^12^. The most serious obstacle to achieving GTR is the difficulty in tumor discrimination and margin delineation during surgery. Various techniques have been employed to distinguish gliomas and demarcate accurate tumor margins. For example, a neuronavigation system based on preoperative magnetic resonance imaging (MRI) has been widely used to perform glioma surgery^13^,^14^,^15^. However, the method often fails to trace tumor margins during the operation due to brain-shift^13^,^16^, and consequently GTR rates are lower as compared with advanced methods such as intraoperative MRI^17^ and fluorescence imaging with 5-aminolevulinic acid (5-ALA)^10^,^18^ or fluorescein sodium^8^. Intraoperative MRI has been used to increase GTR rates, but involves long operation time owing to additional scanning and requires repetitive administration of contrast media such as gadolinium (Gd) chelates^19^. Fluorescence-guided surgery with 5-ALA is a recently adopted method to enable real-time tumor margin discrimination in surgery by means of emission of protoporphyrin IX (ppIX) fluorescence induced by 5-ALA^10^. However, low grade and some grade III glioma tissues do not exhibit visible ppIX fluorescence due to an intact blood brain barrier (BBB) or high ferrochelatase activity. Therefore, this method has not been utilized for World Health Organization (WHO) grade II and III glioma surgeries^20^. Recently, some investigators introduced surface enhanced Raman scattering (SERS) imaging and photoacoustic imaging with contrast media for high grade glioma surgery^21^. However, these methods also do not enable detection of low grade and some grade III gliomas, as an intact BBB prevents such contrast media from extravasating into the tumor. Most up-to-date intraoperative technologies to discriminate and delineate tumor margins for glioma surgery have been suffered from and limited by the followings: (i) brain-shift, (ii) use of contrast agent, or (iii) low sensitivity for low grade glioma and WHO grade III glioma with intact BBB.

Here we present a terahertz reflectometry imaging (TRI) method to tackle the aforementioned limitations. TRI has been applied to brain diseases and cancer diagnosis for over a decade, since it is highly sensitive to water molecules and tissue components^22^,^23^,^24^,^25^,^26^,^27^,^28^. First of all, TRI intrinsically avoids the brain-shift problems of the neuronavigation system because real-time measurement can be performed with portable TRI devices^29^,^30^. Secondly TRI, a label-free imaging method, does not use contrast media. Lastly, TRI is capable of detecting low grade and grade III tumor with intact BBB for the successful GTR.

## Results

### TRI of *ex vivo* human GBM tumorsphere (TS) bearing mouse

**Figure 1:**
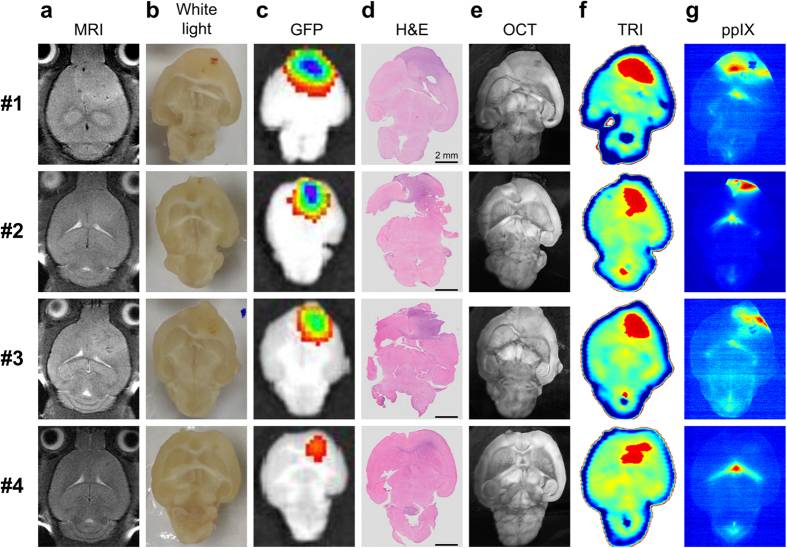
Tumor discrimination of enhanced green fluorescent protein (eGFP)-transfected human GBM tumorsphere (TS) (eGFP^+^ GSC-11) tumor-bearing mice (n = 4) with TRI and multi-modality imaging. (**a**) Axial T2-weighted MRI images in living mouse for validation of tumor growth. (**b**) White light images of the excised brain samples. The tumors were invisible in the white light images as in human malignant gliomas. (**c**) GFP fluorescence images. (**d**) Hematoxylin and eosin (H&E) stained image. Both modalities were used for visualization of tumor regions. (**e**) Optical coherence tomography (OCT) images. These images provide detailed information through the high resolution anatomical structures. Although some regions with reduced scattering may correspond to the tumor region, it is not common feature. (**f**) TRI images with peak-to-peak amplitude of time-domain signals. Relatively high intensity regions (red) in TRI images are well correlated with real tumor regions that are observed in GFP and H&E stained images. We did not determine the precise threshold value in this preclinical experiment. (**g**) 5-ALA-induced ppIX fluorescence images. TRI images showed tumor regions more precisely than ppIX fluorescence images. Strong fluorescence in the center of the ppIX images of the mouse brains is emitted not from tumor but the ventricles.

We conducted TRI of *ex vivo* GBM bearing mouse samples (n = 4). It has been noted that as malignant gliomas grow, they are manifested by a significant reduction of the total lipids and increase in water content^31^,^32^. We therefore hypothesized that TRI may be capable of discriminating glioma from normal brain owing to its high sensitivity to water and lipid content^23^,^33^,^34^. We used an orthotopic xenograft model in which enhanced green fluorescent protein (eGFP)-transfected human GBM TS (eGFP^+^GSC-11) were implanted into BALB/c nude mice (Materials and Methods). The tumor growth was screened with a 9.4T animal MRI, and the mice were sacrificed at 42 days after injection of tumor cells (Fig. 1a and Materials and Methods). 5-ALA solution was injected 2 h before the sacrifice to obtain ppIX fluorescence images, which were compared with TRI images as an existing advanced intraoperative tumor discrimination method. The extracted brain tumor samples were placed on a quartz sample window, and we sequentially obtained white light, TRI, optical coherence tomography (OCT), ppIX fluorescence, GFP fluorescence and Hematoxylin and eosin (H&E) stained images (Materials and Methods). The tumors were invisible in white light images (Fig. 1b), whereas they were clearly visualized in GFP fluorescence and H&E stained images (Fig. 1c,d). OCT images offered detailed anatomical information of brain tissues, but distinct tumor regions could not be visualized (Fig. 1e). The TRI images, in contrast, presented tumor regions with high terahertz (THz) reflection signals compared with normal brain regions (Fig. 1f). The high intensity regions (red, Fig. 1f) in TRI images were better correlated with tumor regions in GFP fluorescence, and H&E stained images, as compared with ppIX images (Fig. 1g). Especially in mouse #4, the fluorescence of tumor regions was not positive in the ppIX fluorescence image due to weak tumor development, which was inferred from the H&E stained image and the low intensity of the GFP fluorescence image; however, the tumor region was well visualized in the TRI image (bottom of Fig. 1).

### TRI for WHO grade II, III, and IV glioma specimens obtained from patients

We performed TRI for human glioma specimens to validate its capability of tumor discrimination from normal brain tissues. Glioma specimens were obtained from surgeries of 14 glioma patients. Tumor grades of the specimen were confirmed as four grade IV, four grade III, and six grade II gliomas by routine pathological examinations (Table 1, Materials and Methods). A neuropathologist diagnosed these tumors according to WHO classification^35^. All TRI images were characterized by the time-domain THz reflectometry system.

**Figure 2:**
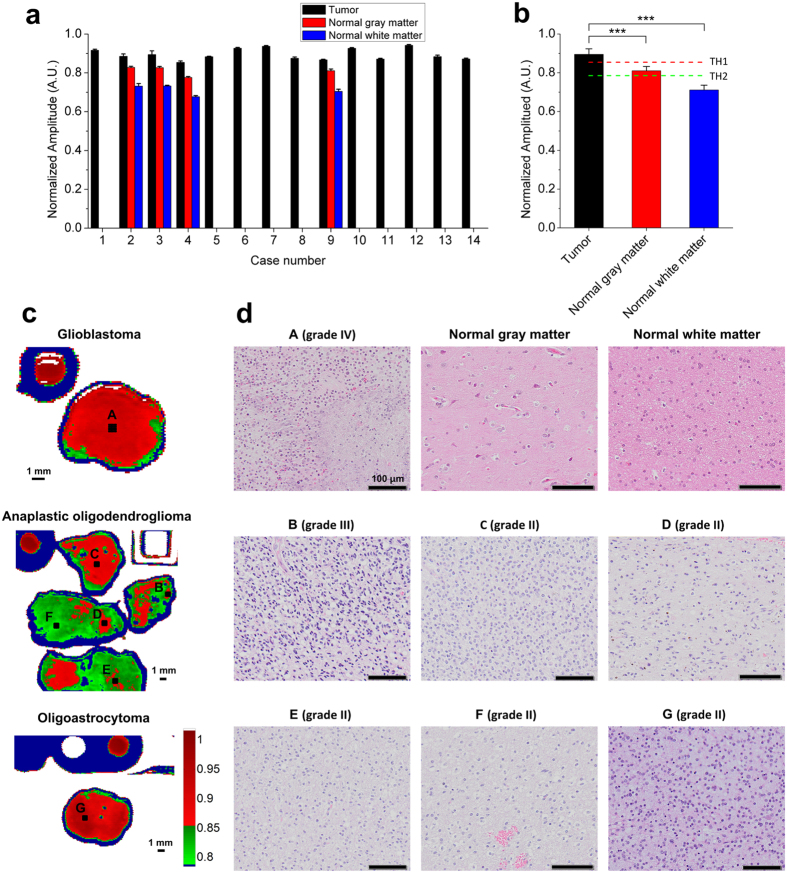
Discrimination of low and high grade of human gliomas with TRI. (**a**) Terahertz parameter (TP) values from regions of interest (ROIs) in tumors (n = 14), normal gray matters (n = 4), and normal white matters (n = 4). (**b**) Quantification of threshold value 1 (TH1) and TH2 (dashed red and green lines, respectively) for tumor discrimination using the data shown in (**a**). Data represent mean ± SD. ***P < 0.001 (Kruskal-Wallis test.) Representative cases of grade IV, III and II gliomas, characterized by (**c**) TRI images and (**d**) H&E stained image. Red regions indicate regions with TP value over TH1. The capitals A–G shown in (**d**) correspond to the ROIs in (**c**).

For a quantitative analysis, we defined a terahertz parameter (TP) as spectrum amplitude ratio at 0.5-THz from the region of interest (ROI). The 0.5-THz is a suitable frequency for biomedical imaging in THz regime^36^. We measured spectrum amplitudes at 0.5-THz in tumor (n = 14), normal gray matter (n = 4), and normal white matter (n = 4) specimens, and then these were normalized to spectrum amplitude at 0.5-THz from each of water reference signals (Fig. 2a, Supplementary Fig. 1). We sampled the normal gray and white matter tissues located in the surgical trajectory to determine the tumor threshold values from the tumor-free specimens. Results were presented as means ± SD (standard deviations), as appropriate. Data were analyzed using SPSS for Windows, Version 12.0 (SPSS Inc., Chicago, IL, USA). Kruskal Wallis test demonstrated that there was a pair having a difference in three groups. Then, the post-hoc test performed by Mann-Whitney test verified the other pair of values the differences in Bonferroni’s method. (Significance level of Bonferroni’s method is 5%/verification number). P values of each tumor, and normal gray and white matters are 0.001. The TP amplitude was 0.8104 AU ± 0.0219 (mean ± standard deviation [S.D.]) in normal gray matter and 0.7114 AU ± 0.0246 in normal white matter; the value in the gray matter was greater than that of the white matter, which may result from the intrinsic difference in their water and lipid contents (Fig. 2b)^37^. We determined two threshold TP values to discriminate tumor from normal brain tissues. The threshold value 1 (TH1) was set to 0.8542 AU, which was determined by mean + 2 S.D. of normal gray matter (dashed red line in Fig. 2b). The red regions denote regions exceeding the TH1 values that correspond to either high grade glioma or a dense tumor region of low grade glioma (Fig. 2a–d). Meanwhile, some regions with TP values smaller than TH1 were pathologically confirmed as grade II gliomas infiltrating into the white matter. It was speculated that when the tumor cells infiltrated to peripheral white matter around dense tumor, the water and lipid contents of diffused tumor region may become similar or lower than that of normal gray matter. The threshold value 2 (TH2) was thus set to be 0.7852 AU, which corresponds to mean + 3 S.D. of normal white matter (dashed green line in Fig. 2b). The green indicates regions in which TPs were smaller than TH1 and larger than TH2 (Fig. 2c-F), corresponding to lower cellularity tumor region than the red regions (Fig. 2c-D,G), even though these green and red regions (Fig. 2c-C–G) represented WHO grade II gliomas histologically. Our results indicate that the green regions in TRI images may represent either low cellularity tumors and diffuse tumors or normal gray matter. Nonetheless, neurosurgeons can judge whether the surgical region is gray matter or white matter based on the information from the surgical microscope, their anatomical knowledge, preoperative MRI and neuronavigation. Therefore, the green region in TRI images would provide useful information to neurosurgeons about low grade or diffused tumor regions in surgery. It is worth noting that low grade gliomas, where ppIX fluorescence has been scarcely expressed, could be well discriminated by TRI. Although the four tumor specimens including ROIs B-E in Fig. 2c were obtained from the same patient, 5-ALA-induced ppIX fluorescence was only expressed at specimens including ROIs B and C, but not in other specimens; in contrast, all tumors were discriminated in TRI images (Fig. 2c–d). We assume that our TRI image results reflect a compositional change associated with increased water content and decreased lipid content and this change accompanied not only high grade but also low grade gliomas^32^,^38^. As a result, we could find the presence of tumor in all cases by TRI images.

**Figure 3:**
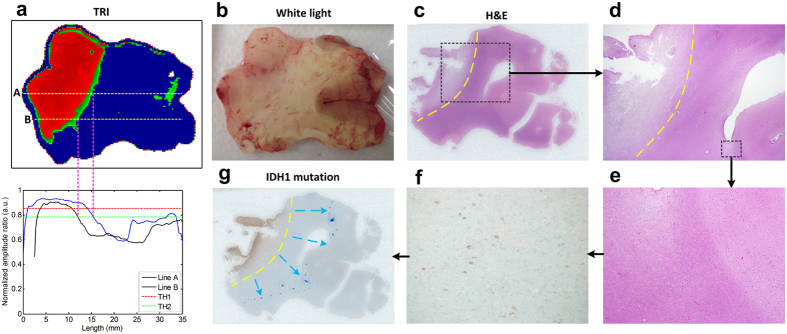
Delineation of tumor margin with TRI. (**a**) TRI image of a WHO grade II glioma specimen (case 6). The red and green regions denote tumor regions exceeding TH1 and TH2. The graph below TRI image shows the display for terahertz parameter value along the dashed line A and B. The green region on right of specimen is not tumor because the region is located in gray matter. (**b**) White light image of the specimen. (**c**) Tumor margins (yellow dashed line) determined by H&E stained image. The result agreed well to the tumor margin detected by TRI image. (**d**) Magnified H&E image (x12.5) of the black dashed box region in (**c**). (**e**) A further magnified image (x40) of black dashed box region in d, in which tumor cells could not be identified. (**f**) However, immunohistochemistry showed the presence of IDH1 mutated cells (brown) on the same region of (**e**), which have been known to be indicated glioma cells. A further magnified immunohistochemistry image (x400) of some region in (**e**). (**g**) Based on the IDH1 mutation stained image, the tumor margins (blue dots) should be re-delineated beyond the margins determined by H&E stained image.

We then validated the tumor margin delineation ability of TRI. The wavelength of terahertz electromagnetic waves is a few hundred-μm, so TRI may be more suitable as a macroscopic imaging tool of gliomas. While TRI may not be able to delineate tumor margin at the cellular level, it has the potential to macroscopically delineate foci-glioma margins. Indeed, our macroscopic delineation of the tumor margins were correlated with the pathologically determined tumor margins with the H&E stained image. Red and green tumor regions over TH1 and TH2 in the TRI image of low grade glioma specimen (case 6) were well correlated with the tumor margin pathologically determined with the H&E stained image (Fig. 3a–c). However, a few tumor cells existed outside of the margin of the foci-tumor region, and the diffused tumor cells were confirmed in an immunochemistry stained image (isocitrate dehydrogenase 1 [IDH1, 1/80, clone H09, Dianova, Germany] stain, Materials and Methods) and not in a H&E stained image (Fig. 3c–f). The precise tumor margins determined with the immunochemistry stained image was found to be further extended than those determined with the TRI image and the H&E image (Fig. 3g). Therefore, when a neurosurgeon practically uses this method during glioma surgery, the neurosurgeon would need to apply safety margin that could be determined by further investigations or other complementary microscopic imaging tools to complete the glioma resection.

### TRI of *in vivo* live mouse tumor model

**Figure 4:**
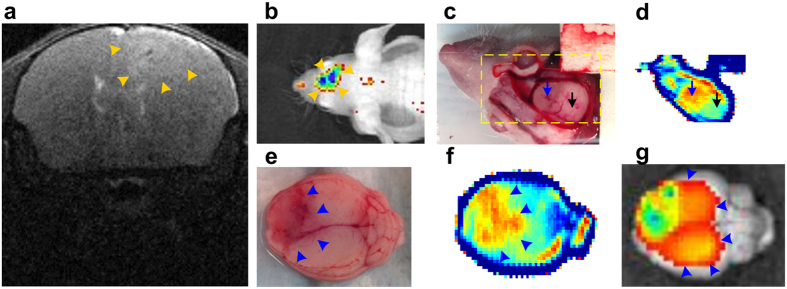
*In vivo* tumor detection in a mouse via TRI. (**a**) T2-weighted coronal MRI image. (**b**) *In vivo* GFP fluorescence image. The presence of tumor was screened by the MRI and fluorescence images. Yellow-orange arrowheads indicate the assumed tumor region. The *in vivo* experiment was performed after the tumor developed to the brain surface. (**c**) White light image of exposed brain tumor that was in contact with the quartz window. Yellow dashed box indicates region of measured TRI image. (**d**) *In vivo* peak-to-peak TRI image. Tumor regions (blue arrow) were well discriminated from normal brain regions (black arrow) in the live animal. (**e–g**) White light, TRI, and GFP fluorescence images of extracted whole brain, respectively. The gross tumor regions, determined by each image (blue arrowheads), were well discriminated in the TRI image and even in the white light image (violet arrow). The tumor region in GFP fluorescence image (**g**) seems to be broader than that in THz image (**f**). The discrepancy may be accounted for by that GFP fluorescence image results in part from the diffusive fluorescence signal from the tumors deep in the tissue (Supplementary Figs 3 and 4).

Finally, we assessed TRI for *in vivo* tumor detection in a living mouse with eGFP^+^GSC-11 cell xenotransplantation (n = 1). We tracked the tumor growth with 9.4T MRI and GFP fluorescence imaging (Fig. 4a,b). After the tumor had grown to the outer surface of brain, we made a cranial window and exposed the brain tumor to obtain the TRI image (Supplementary Fig. 2). The tumor showed a distinctively high intensity signal (red) that was well discriminated from adjacent normal brain tissue in the *in vivo* TRI image (blue and black arrows in Fig. 4c,d). We re-confirmed the tumor detection after the whole brain was extracted from the mouse. The high intensity regions in TRI image of extracted brain surface corresponded well to the tumor regions that were later confirmed by GFP fluorescence imaging (Fig. 4e–g).

## Discussion

We describe a highly sensitive TRI for low and high grade glioma identification without exogenous contrast agents. TRI is an imaging technique, implementable to the biomedical field. Because TRI signal is affected by the molecular dynamics of water and the differences in the refractive indices between water and lipids, the resultant images reflect high sensitivity to water and lipid content in tissues^33^,^39^. Glioma is characterized by an increased level of water and smaller lipid content in contrast to the adjacent normal brain^31^,^32^. Therefore, TRI may serve as a high-sensitivity detector for glioma, allowing neurosurgeons to ‘see’ tumor regions without the use of contrast agents during glioma surgery. Several papers reported on the possibility of glioma detection using TRI^23^,^40^,^41^. However they used only fresh rat or paraffin-embedded samples. In this paper, we performed TRI imaging in not only fresh mouse samples but also fresh human specimens. We further carried out *in vivo* TRI imaging of a tumor-bearing mouse model. Through the experiments, we validated the feasibility of TRI for glioma detection with preclinical, clinical and *in vivo* settings in only an article.

TRI exhibits distinct advantages compared with conventional intraoperative surgical methods. Firstly, TRI, in a form of handheld device, would be intrinsically obviated from brain-shift challenges that surgeons face frequently in using the brain neuronavigation system. Handheld TRI devices have been already developed^29^,^30^, so the neurosurgeons could utilize these tools to locate tumors during operation. Secondly, this method has the potential to provide a real-time display for the tumor detection at surgery^42^. Thirdly, TRI is a label-free method, so it is unrestrained from the various adverse effects of contrast agent: photo-bleaching, restricted photo retention time, patient hypersensitivity to light at ppIX fluorescence-guided surgery method with 5-ALA, and the adverse effects produced by repeated gadolinium contrast administration at intraoperative MRI^19^. Finally, this method could discriminate not only high grade gliomas but also low grade gliomas from normal brain tissues, whereas ppIX fluorescence imaging is only effective in high-grade gliomas.

Recent advances in Raman spectroscopy made it possible to discriminate high and low grade gliomas with a sensitivity and a specificity of 93% and 91% respectively^43^,^44^. Raman methods also use the intrinsic optical properties of tissues to characterize the decreased lipid content in gliomas. Therefore, the results from the Raman technique could indirectly imply TRI’s high feasibility in the clinical glioma surgery application.

Though Raman imaging has remarkable capability to detect gliomas, it has several obstacles to practically be employed in glioma surgery such as low signal to noise ratio and long measurement time. It is difficult to compare TRI directly with Raman imaging, because there are differences in operating principles and implementations. With regard to spatial resolution, TRI is generally macroscopic, whereas Raman imaging is microscopic^24^,^44^,^45^. These features may be combined to provide complementary information for the complete resection of gliomas. For example, neurosurgeons may preferentially remove most of the glioma through TRI-validated images where the macroscopic tumor margins are visualized, and then they can completely remove residual glioma tissue in the periphery of the tumor through Raman-validated images where diffusely invasive brain cancer cells are identified at cellular resolution.

The penetration depth of THz waves in tissues is rather limited (generally under 500-μm) due to the high absorption by water^46^. As such, TRI could detect tumor only when the tumor is exposed on the outer surface of the brain. However, we do not consider this as a serious limitation because ppIX fluorescence-guided surgery is also limited by a short penetration depth but is very helpful to neurosurgeons.

In some regions of our three human glioma cases (cases 8, 11 and 13), false-positives over TH1 were identified although the regions did not possess tumor tissue on histological examination. High-reflection THz signals larger than the TH1 should be considered abnormal despite scarce or absent tumor cells. Further investigation on this observation will be performed with a larger set of clinical data. As previously mentioned, TRI is a macroscopic method, so diffused tumor or peripheral foci-tumor may be expressed as low-signal regions below TH2. This limitation may be overcome by simultaneous use of TRI with a complementary microscopic method such as Raman imaging.

Today, neurosurgeons mobilize various intraoperative technologies such as the neuronavigation system, intraoperative MRI, 5-ALA-induced ppIX fluorescence imaging, and ultrasonic systems for complete tumor resection. Our TRI method enables the neurosurgeon to detect during surgery not only high grade gliomas, but also low grade gliomas with intact BBB involving increased water and decreased lipid content. In the foreseeable future, TRI can mitigate the bottleneck of incomplete tumor resection rate in low grade gliomas that are underestimated by ppIX fluorescence imaging. We expect this method will be able to substantially contribute to successful glioma resections and lead to better outcomes for glioma patients in the future.

## Materials and Methods

### Study design

Conventional imaging methods for glioma surgery have been limited by the following: (i) brain-shift, (ii) use of contrast agent, or (iii) low sensitivity to low grade glioma. The main aim of this study was to introduce and demonstrate the potential feasibility of TRI for glioma surgery that enables to overcome current key limitations for intraoperative detection of high and low grade gliomas. It has been known that malignant gliomas are manifested by a significant reduction of the total lipids and increase in water content. We therefore hypothesized that TRI may be capable of discriminating glioma from normal brain due to its high sensitivity to water and lipid content. First, we conducted preclinical experiments with *ex vivo* xenotransplantation brain tumor models (n = 4) to verify our hypothesis. We compared TRI images with multi-modality images including standard H&E images. Next, we evaluated TRI of fresh WHO grade II, III, and IV glioma specimens obtained from human patients (n = 14). Finally, we demonstrated that TRI is a viable technique for *in vivo* glioma detection using an *in vivo* xenotransplantation brain tumor model (n = 1).

### Lentiviral vector transduction and expression

Green fluorescent protein (GFP) stably expressing GSC11 cells (GSC11-GFP) were generated by growing GSC11 cells in complete medium and then applying GFP-expressing lentiviral supernatants. Polybrene (Sigma, Dorset, UK) was added to a final concentration of 8 μg/ml and incubated with cells for 18 h. After infection, the cells were placed in fresh growth medium and cultured in a standard manner. Cells were treated with 1 mg/ml puromycin (Life Technologies Korea, Seoul, Republic of Korea) to eliminate uninfected cells and generate stable GSC11-GFP. GSC11-GFP was isolated for use in further experiments by fluorescence activated cell sorting (FACS).

### Mouse care and information

Five-week-old male athymic Balb/c nude mice (Orient Bio, Seongnam-Si, Republic of Korea) were used for tumor xenograft experiments. Mice were retained in micro-isolator cages under sterile conditions and observed for at least 1 week before study initiation to ensure proper health. Temperature, lighting and humidity were controlled centrally. All experimental procedures were carried out in accordance with the guidelines Institutional Animal Care and Use Committees (IACUC) which were approved by Yonsei University College of Medicine Institutional Animal Care and Use Committee. The mice were anesthetized with a solution of Zoletil (30 mg/kg; Virbac Korea, Seoul, Republic of Korea) and xylazine (10 mg/kg; Bayer Korea, Seoul, Republic of Korea) delivered intraperitoneally. After the holes were drilled on the mouse skull using a 26-gauge needle, GSC11-GFP cells (1 × 10^6^) were implanted directly into right frontal lobe of nude mice using Hamilton syringe (Dongwoo Science Co., Seoul, Republic of Korea) at the depth of 4.5 mm. Cells were simultaneously injected into five mice using a multiple microinfusion syringe pump (Harvard Apparatus, Holliston, MA, USA) at a rate of 0.5 μl/min as previously described^47^,^48^.

### 9.4T animal MRI

We performed *in vivo* MR imaging experiments with a 9.4T animal MRI instrument with a Bruker animal coil (RF SUC 400 1H M-BR-LIN ROAD, Bruker Medical Systems, Germany). The sequence parameters were adopted as follows: Echo = 1, TR = 2500 ms, TE = 22.2 ms, FA = 180 deg, TA = Oh2m5s0ms, NEX = 1, FOV = 2.50/1.80 cm.

### TRI system

To measure the THz signal, we used a homemade THz reflectometric imaging system with photoconductive antennas that were driven by femto-second laser pulses to generate and detect terahertz pulses. We used four off-axis parabolic reflectors to collimate and focus THz pulses on *ex vivo* and *in vivo* samples and guide reflected THz pulses to the detector. The extracted mouse tumor samples and glioma specimens from patients were placed on a quartz sample window, and then we mounted the sample window on a computer-controlled x-y translation stage which was used to raster scan. The focused beam diameter of THz pulses was 0.8-mm at 0.5 THz, and THz signals were measured with 20 Hz and 250-μm scanning resolution. A more detailed description is presented in our previous report^45^. We obtained TRI images for *ex vivo* and *in vivo* mouse samples using peak-to-peak amplitude extracted from reflection THz signals.

### Combined Optical Coherence Tomography (OCT) and ppIX fluorescence imaging system

Our OCT system was implemented based on optical frequency domain imaging as described in [Yun *et al*. Opt. Express, 11: 2953–2963 (2003); Yun *et al*. Nature Medicine, 12: 1429–1433 (2006)]. A 1.31-μm wavelength-swept laser (SS-1310, Axsun Technologies Inc., USA) was employed as a light source, which provided a sweep range of 110 nm, repetition rate of 50 kHz, and average power of ~20 mW. Light from the laser was first directed to a 90/10 fiber coupler, and 10% of the light was tapped and detected with a fast InGaAs photodetector through a narrowband fixed-wavelength filter, serving as a trigger for signal acquisition. The k-clock from the laser was directly connected to a digitizer for signal sampling. Ninety percent of the remaining light was directed to the OCT interferometer.

In the OCT sample arm, we combined ppIX fluorescence imaging setup to perform a direct comparison of the tumor regions measured by TRI and OCT against the regions by ppIX fluorescence imaging. A 405-nm laser diode (FL laser) was employed as a fluorescence excitation light source. The light from the laser was combined with the 1.3-μm OCT probe light through the dichroic mirrors DM1 (47267, Edmund Optics, USA) and DM2. The combined light was then directed to a specimen through the same galvanometric scanners (GM) and illumination optics described above. This arrangement enabled both OCT and fluorescence images to share the same field of view, facilitating direct image comparison. The beam size of the 405-nm excitation light on the specimen plane was measured to be 80 μm. The backscattered OCT light from the sample was re-coupled to the fiber and its interference with reflection from a reference mirror was detected by a high-speed balanced detector (Thorlabs, Inc., PDB410C, New Jersey, USA). For the fluorescence signal, the fluorescence emission from the specimen was collected by the focusing lens, reflected by the dichroic mirror DM3 (NFD-01-633, Semrock, USA), and subsequently detected with a large-area photodetector (PDA100A-EC-Si, Thorlabs, USA) through a lens.

### GFP fluorescence imaging

We performed ex vivo fluorescence imaging using green fluorescence protein. The fluorescence recovery profiles in eGFP^+^ GSC-11 tumor-bearing mice were imaged by positioning mice on an animal plate, heated to 37 °C, in the IVIS spectrum system (Caliper Life Science, Hopkinton, MA, USA) as per the manufacturer’s instructions. Excitation and emission spots were raster-scanned over the selected region of interest (ROI) to generate emission wavelength scans, wavelength of fluorescence were 488 nm and 520 nm respectively for *ex vivo* samples. All data, including whole body and two-dimensional (2D) slice images, were calculated using the ROI function of the Analysis Workstation software.

### Patient population

Fourteen patients with WHO grade II, III, and IV glioma, including 4 grade IV, 4 grade III, and 6 grade II patients, treated at our institution between February 2014 and December 2014 were included in this study (Table 1). All patients were histologically diagnosed and graded by neuro-pathologists according to 2007 WHO classification criteria. Informed consent was provided according to the Declaration of Helsinki. All patients provided written informed consent, and the study was approved by the Institutional Review Boards of Severance Hospital, Yonsei University College of Medicine. All experiments were conducted in accordance with principles expressed in the Declaration of Helsinki or other relevant guidelines and regulations. O-6-methylguanine-DNA methyltransferase (MGMT) promotor methylation and isocitrate dehydrogenase (IDH)-1 mutations were assessed by polymerase chain reaction (PCR) and immunohistochemistry (IHC). Epidermal growth factor receptor (EGFR) amplification and loss of heterozygosity (LOH) at chromosomes 1p and 19q were evaluated by fluorescent *in situ* hybridization (FISH). P53 and ki-67 were examined by IHC.

### IDH1 Immmunohistochemistry (IHC)

With a representative section, we performed IHC with a VentanaBenchMark XT autostainer (Ventana Medical System, Inc. Tucson, AZ) according to the protocol. The antibody used was anti-human IDH1 R132H mouse monoclonal (Clone H09L, 1:80 dilution, Dianova, Hamburg, Germany). A neuropathologist (S.H. Kim) reviewed the immunohistochemical and histologic findings without information of IDH1 mutation status assessed by other methods. When the cytoplasmic expression of the IDH1 R132H was identified in glioma cells, we regarded those cases as “mutant” or “positive”.

## Additional Information

**How to cite this article**: Ji, Y. B. *et al*. Terahertz reflectometry imaging for low and high grade gliomas. *Sci. Rep.* **6**, 36040; doi: 10.1038/srep36040 (2016).

**Publisher’s note:** Springer Nature remains neutral with regard to jurisdictional claims in published maps and institutional affiliations.

## Supplementary Material

Supplementary Information

## Figures and Tables

**Table 1:** Summary of patient pathological information.

Case	Age	Sex	Pathology	WHO grade	5-ALA	IDH1	MGMT	1p/19q co-deletion	Ki-67 L.I.
1	57	F	Glioblastoma	IV	+	Wild	Unmethylated	No	50%
2	55	M	Glioblastoma	IV	+	Wild	Methylated	No	5–7%
3	67	M	Glioblastoma	IV	+	Wild	Methylated	Yes	15–20%
4	62	F	Glioblastoma	IV	+	Wild	Unmethylated	No	15–20%
5	46	M	Anaplastic oligodendroglioma	III	+	Mutant	Methylated	No	3–4%
6	34	F	Mixed oligoastrocytoma	II	NA	Mutant	Unmethylated	No	3–4%
7	51	F	Oligodendroglioma	II	NA	Mutant	Methylated	Yes	3–4%
8	40	F	Diffuse astrocytoma	II	NA	Wild	Unmethylated	No	2–3%
9	56	F	Anaplastic astrocytoma	III	+	Wild	Unmethylated	No	5–6%
10	41	F	Anaplastic astrocytoma	III	−	Wild	Unmethylated	No	50%
11	36	M	Anaplastic astrocytoma	III	NA	Mutant	Methylated	Yes	20–25%
12	51	F	Oligoastrocytoma	II	NA	Mutant	Methylated	No	2–3%
13	38	M	Oligoastrocytoma	II	NA	Mutant	Unmethylated	No	5%
14	41	M	Oligodendroglioma	II	NA	Mutant	Methylated	Yes	3–4%

WHO: World Health Organization; 5-ALA: 5-aminolevulinic acid; IDH1: isocitrate dehydrogenase 1; MGMT: O6-methylguanine-methyltransferase; 1p/19q: chromosomal co-deletions that are characteristic of oligodendrogliomas; Ki-67 L.I.: labeling index associated with proliferation; NA: not available.

## References

[b1] Ostrom Q. T. . CBTRUS statistical report: primary brain and central nervous system tumors diagnosed in the United States in 2007–2011. Neuro-oncology 16 Suppl 4, iv1–63 (2014). 10.1093/neuonc/nou223 PMC4193675 25304271

[b2] Stupp R. . Radiotherapy plus concomitant and adjuvant temozolomide for glioblastoma. The New England journal of medicine 352, 987–996 (2005). 15758009 10.1056/NEJMoa043330

[b3] Takahashi Y. . Prognostic value of isocitrate dehydrogenase 1, O6-methylguanine-DNA methyltransferase promoter methylation, and 1p19q co-deletion in Japanese malignant glioma patients. World J Surg Oncol 11, 284 (2013). 24160898 10.1186/1477-7819-11-284 PMC3874767

[b4] Chang E. F. . Multiinstitutional validation of the University of California at San Francisco Low-Grade Glioma Prognostic Scoring System. Clinical article. J Neurosurg 111, 203–210 (2009). 19267536 10.3171/2009.2.JNS081101

[b5] Phuphanich S., Ferrall S. & Greenberg H. Long-term survival in malignant glioma. Prognostic factors. J Fla Med Assoc 80, 181–184 (1993). 8387566

[b6] Winger M. J., Macdonald D. R. & Cairncross J. G. Supratentorial anaplastic gliomas in adults. The prognostic importance of extent of resection and prior low-grade glioma. J Neurosurg 71, 487–493 (1989). 2552044 10.3171/jns.1989.71.4.0487

[b7] Almenawer S. A. . Biopsy versus partial versus gross total resection in older patients with high-grade glioma: a systematic review and meta-analysis. Neuro-oncology 17, 868–881 (2015). 25556920 10.1093/neuonc/nou349 PMC4483123

[b8] Chen B. . Gross total resection of glioma with the intraoperative fluorescence-guidance of fluorescein sodium. Int J Med Sci. 9, 708–714 (2012). 23091408 10.7150/ijms.4843 PMC3477680

[b9] Abrey L. E. Gross total resection of low-grade glioma in adults. Curr Neurol Neurosci Rep. 9, 181–182 (2009). 19348705 10.1007/s11910-009-0027-4

[b10] Stummer W. . Fluorescence-guided surgery with 5-aminolevulinic acid for resection of malignant glioma: a randomised controlled multicentre phase III trial. The lancet oncology 7, 392–401 (2006). 16648043 10.1016/S1470-2045(06)70665-9

[b11] Grier J. T. & Batchelor T. Low-grade gliomas in adults. Oncologist 11, 681–693 (2006). 16794247 10.1634/theoncologist.11-6-681

[b12] Sanai N. & Berger M. S. Glioma extent of resection and its impact on patient outcome. Neurosurgery 62, 753–764; discussion 264–756 (2008). 18496181 10.1227/01.neu.0000318159.21731.cf

[b13] Nimsky C. . Preoperative and intraoperative diffusion tensor imaging-based fiber tracking in glioma surgery. Neurosurgery 61, 178–185; discussion 186 (2007). 18813171 10.1227/01.neu.0000279214.00139.3b

[b14] Roessler K. . Frameless stereotactic guided neurosurgery: clinical experience with an infrared based pointer device navigation system. Acta Neurochir (Wien) 139, 551–559 (1997). 9248590 10.1007/BF02750999

[b15] Willems P. W., van der Sprenkel J. W., Tulleken C. A., Viergever M. A. & Taphoorn M. J. Neuronavigation and surgery of intracerebral tumours. J Neurol. 253, 1123–1136 (2006). 16988793 10.1007/s00415-006-0158-3

[b16] Trantakis C. . Investigation of time-dependency of intracranial brain shift and its relation to the extent of tumor removal using intra-operative MRI. Neurol Res. 25, 9–12 (2003). 12564118 10.1179/016164103101200923

[b17] Senft C. . Intraoperative MRI guidance and extent of resection in glioma surgery: a randomised, controlled trial. The lancet oncology 12, 997–1003 (2011). 21868284 10.1016/S1470-2045(11)70196-6

[b18] Schatlo B. . Outcomes after combined use of intraoperative MRI and 5-aminolevulinic acid in high-grade glioma surgery. Neuro-oncology (2015). 10.1093/neuonc/nov049 PMC4633924 25858636

[b19] Ludemann L., Hamm B. & Zimmer C. Pharmacokinetic analysis of glioma compartments with dynamic Gd-DTPA-enhanced magnetic resonance imaging. Magn Reson Imaging 18, 1201–1214 (2000). 11167040 10.1016/s0730-725x(00)00223-x

[b20] Roessler K., Becherer A., Donat M., Cejna M. & Zachenhofer I. Intraoperative tissue fluorescence using 5-aminolevolinic acid (5-ALA) is more sensitive than contrast MRI or amino acid positron emission tomography ((18)F-FET PET) in glioblastoma surgery. Neurol Res. 34, 314–317 (2012). 22449387 10.1179/1743132811Y.0000000078

[b21] Kircher M. F. . A brain tumor molecular imaging strategy using a new triple-modality MRI-photoacoustic-Raman nanoparticle. Nat Med. 18, 829–834 (2012). 22504484 10.1038/nm.2721 PMC3422133

[b22] Png G. M., Flook R., Ng B. W. H. & Abbott D. Terahertz spectroscopy of snap-frozen human brain tissue: an initial study. Electron Lett. 45, 343–344 (2009).

[b23] Oh S. J. . Study of freshly excised brain tissues using terahertz imaging. Biomed Opt Express 5, 2837–2842 (2014). 25136506 10.1364/BOE.5.002837 PMC4133010

[b24] Ji Y. B. . Feasibility of terahertz reflectometry for discrimination of human early gastric cancers. Biomed Opt Express 6, 1398–1406 (2015). 25909023 10.1364/BOE.6.001398 PMC4399678

[b25] Reid C. B. . Terahertz pulsed imaging of freshly excised human colonic tissues. Phys Med Biol. 56, 4333–4353 (2011). 21709342 10.1088/0031-9155/56/14/008

[b26] Fitzgerald A. J. . Terahertz pulsed imaging of human breast tumors. Radiology 239, 533–540 (2006). 16543586 10.1148/radiol.2392041315

[b27] Wallace V. P. . Terahertz pulsed imaging of basal cell carcinoma *ex vivo* and *in vivo*. Brit J Dermatol. 151, 424–432 (2004). 15327550 10.1111/j.1365-2133.2004.06129.x

[b28] Woodward R. M. . Terahertz pulse imaging of *ex vivo* basal cell carcinoma. The Journal of investigative dermatology 120, 72–78 (2003). 12535200 10.1046/j.1523-1747.2003.12013.x

[b29] Ji Y. B., Lee E. S., Kim S. H., Son J. H. & Jeon T. I. A miniaturized fiber-coupled terahertz endoscope system. Opt Express 17, 17082–17087 (2009). 19770926 10.1364/OE.17.017082

[b30] Parrott E. P., Sy S. M., Blu T., Wallace V. P. & Pickwell-Macpherson E. Terahertz pulsed imaging *in vivo*: measurements and processing methods. Journal of biomedical optics 16, 106010 (2011). 22029357 10.1117/1.3642002

[b31] Yates A. J., Thompson D. K., Boesel C. P., Albrightson C. & Hart R. W. Lipid composition of human neural tumors. Journal of lipid research 20, 428–436 (1979). 458265

[b32] Kohler M., Machill S., Salzer R. & Krafft C. Characterization of lipid extracts from brain tissue and tumors using Raman spectroscopy and mass spectrometry. Analytical and bioanalytical chemistry 393, 1513–1520 (2009). 19153721 10.1007/s00216-008-2592-9

[b33] Reid C. Spectroscopic methods for medical diagnosis at terahertz wavelengths. In Department of Medical Physics and Bioengineering Vol. Doctor of philosophy 92–94 (University College London, 2009).

[b34] Grootendorst M. R., Cariati M., Ashworth P. C., Fitzgerald A. J., Purushotham A. & Wallace V. P. Application of terahertz technology to breast cancer. In Terahertz biomedical science & technology (ed. Son J.-H.) 312–314 (CRC Press, 2014).

[b35] Louis D. N., Ohgaki H., Wiestler O. D. & Cavenee W. K. WHO classification of tumours of the central nervous system (Lyon: International Agency for Research on Cancer, 2007). 10.1007/s00401-007-0243-4 PMC1929165 17618441

[b36] Taylor Z. D. . THz Medical Imaging: *in vivo* Hydration Sensing. Ieee T Thz Sci Techn. 1, 201–219 (2011). 10.1109/TTHZ.2011.2159551 PMC4467694 26085958

[b37] O’Brien J. S. & Sampson E. L. Lipid composition of the normal human brain: gray matter, white matter, and myelin. Journal of lipid research 6, 537–544 (1965). 5865382

[b38] Bartha R., Megyesi J. F. & Watling C. J. Low-grade glioma: correlation of short echo time 1H-MR spectroscopy with 23Na MR imaging. AJNR. American journal of neuroradiology 29, 464–470 (2008). 18238848 10.3174/ajnr.A0854 PMC8118860

[b39] Ashworth P. C. . Terahertz pulsed spectroscopy of freshly excised human breast cancer. Opt Express 17, 12444–12454 (2009). 19654646 10.1364/oe.17.012444

[b40] Meng K. . Terahertz pulsed spectroscopy of paraffin-embedded brain glioma. Journal of biomedical optics 19, 077001 (2014). 25003757 10.1117/1.JBO.19.7.077001

[b41] Yamaguchi S. . Brain tumor imaging of rat fresh tissue using terahertz spectroscopy. Scientific Reports 6, 30124 (2016). 27456312 10.1038/srep30124 PMC4960480

[b42] Yee D. S. . High-speed terahertz reflection three-dimensional imaging using beam steering. Opt Express 23, 5027–5034 (2015). 25836537 10.1364/OE.23.005027

[b43] Jermyn M. . Intraoperative brain cancer detection with Raman spectroscopy in humans. Science translational medicine 7, 274ra219 (2015). 10.1126/scitranslmed.aaa2384 25673764

[b44] Ji M. . Rapid, label-free detection of brain tumors with stimulated Raman scattering microscopy. Science translational medicine 5, 201ra119 (2013). 10.1126/scitranslmed.3005954 PMC3806096 24005159

[b45] Ji Y. B. . Terahertz spectroscopic imaging and properties of gastrointestinal tract in a rat model. Biomed Opt Express 5, 4162–4170 (2014). 25574429 10.1364/BOE.5.004162 PMC4285596

[b46] Pickwell E. & Wallace V. P. Biomedical applications of terahertz technology. J Phys D Appl Phys 39, R301–R310 (2006).

[b47] Kong B. H. . Isolation of glioma cancer stem cells in relation to histological grades in glioma specimens. Child Nerv Syst. 29, 217–229 (2013). 10.1007/s00381-012-1964-9 23143002

[b48] Lal S. . An implantable guide-screw system for brain tumor studies in small animals. Journal of Neurosurgery 92, 326–333 (2000). 10659021 10.3171/jns.2000.92.2.0326

