# Supplementary material for: Terahertz reflectometry imaging for low and high grade gliomas
Source: Sci Rep. 2016 Oct 26;6:36040. doi: 10.1038/srep36040 (PMC5080552; doi:10.1038/srep36040)
Supplement: Supplementary Information [file srep36040-s1.pdf]

Terahertz reflectometry imaging for low and high grade gliomas

Young Bin Ji[1,9], Seung Jae Oh[1,9], Seok-Gu Kang[2,9], Jung Heo[3,9], Sang-Hoon Kim[1,4], Yuna Choi[1], Seungri Song[3], Hye Young Son[5], Se Hoon Kim[6], Ji Hyun Lee[2], Seung Joo Haam[7], Yong Min Huh[1,8], Jong Hee Chang[2,*], Chulmin Joo[3,*] and Jin-Suck Suh[1,8,*]

[1]YUHS-KRIBB Medical Convergence Research Institute, Yonsei University College of Medicine, Seoul, Republic of Korea.

[2]Department of Neurosurgery, Brain Tumor Center, Brain Research Institute, Severance Hospital, Yonsei University College of Medicine, Seoul, Republic of Korea.

[3]Department of Mechanical Engineering, Yonsei University, Seoul, Republic of Korea.

[4]Applied Electromagnetic Wave Research Center, Korea Electrotechnology Research Institute, Ansan, Republic of Korea.

[5]Severance Biomedical Science Institute, Severance Hospital, Yonsei University College of Medicine, Seoul, Republic of Korea.

[6]Department of Pathology, Severance Hospital, Yonsei University College of Medicine, Seoul, Republic of Korea.

[7]Department of Chemical and Biomolecular Engineering, Yonsei University, Seoul, Republic of Korea.

[8]Department of Radiology, Severance Hospital, Yonsei University College of Medicine, Seoul, Republic of Korea.

[9]These authors contributed equally to this work.

*These authors jointly directed this work. Correspondence should be addressed to J.S.S. (jss@yuhs.ac) or C. J. (cjoo@yonsei.ac.kr) or J.H.C. (changjh@yuhs.ac).

Supplementary information

1. **Extraction of the Terahertz (THz) parameter (TP) from measured reflection-THz signals.**

Using our TRI system, we were able to obtain time-domain signals at each measurement point (**Online method, Supplementary Fig. 1a,b**). And we also easily obtained amplitude spectra at each point by performing fast Fourier transform to time-signals (**Supplementary Fig. 1c**). The TP is:

$$\mathrm{TP} = \left.\frac{E_{specimen}(\omega)}{E_{water}(\omega)}\right|_{\omega = 0.5\,THz}$$

where ω is frequency, $E_{specimen}(\omega)$and $E_{water}(\omega)$ are reflection-spectrum amplitude from quartz/specimen and quartz/water interfaces at specific frequency (ω=0.5 THz). We measured water signals as reference signals in every experiment and the TP has a normalization-term to prevent experimental error occurring from the THz imaging system (**Supplementary Fig. 1d**).

We chose 25 points in each case (n=14) as regions of interest (ROI) of tumor for determination of threshold (**Supplementary Fig. 1a**). All of the ROIs of tumor were possessed tumor tissues on histological examination. And we chose each 25 points ROIs of normal gray & white matter in four cases where the tumor were not present in normal brain specimens on histological examination.

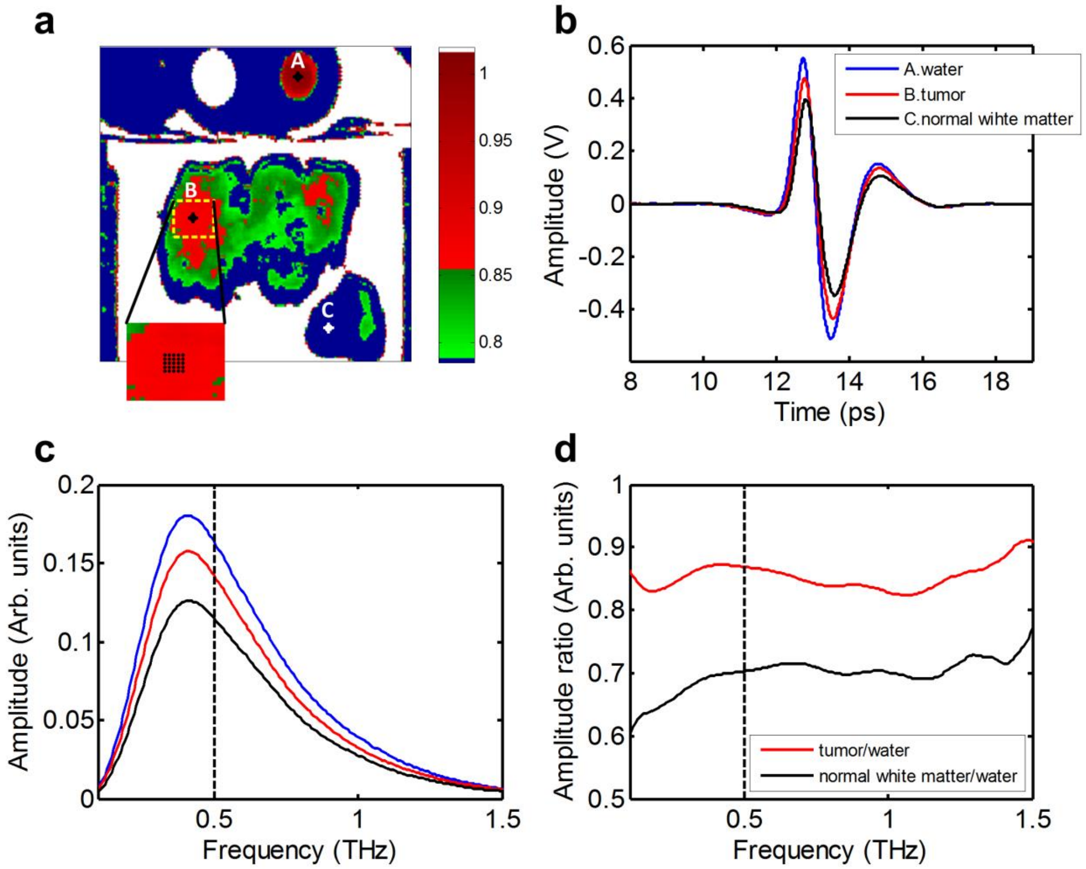

Supplementary Figure 1. TP extraction from raw THz data. (a) The TRI image of glioma & normal brain specimen (case 9) using TP values. A, B, C indicates ROIs of water, tumor and normal white matter respectively. (b) Time-domain THz signals from water, tumor and normal white matter. (c) Spectra of the signals in (b). (d) The TP values of tumor and normal white matter between 0.1 and 1.5THz. The spectra were normalized by spectra of water.

2. ***In vivo* TRI experiment**.

A same tumor model used in previous *ex vivo* experiments was used for the *in vivo* experiment. We performed the *in vivo* experiment after tumor was grown to surface of brain (**Fig. 4a**). We were anesthetized the mouse for this *in vivo* experiment in the same way as the method described in Online methods. Firstly, we marked the tumor side on rump of the

mouse (**Supplementary Fig. 2a**), and disinfected overall head with povidone-iodine before removing the head skin (**Supplementary Fig. 2b**). Secondly, we carefully cut the head skin and removed the skull using a disinfected micro-motor handpiece (SDE-H37L) without vascular injuries (**Supplementary Fig. 2c-e**). Thirdly, we made the exposed brain keep contact to quartz window with adhesive tape for measuring THz signals. Finally, we obtained the THz signals reflected from the brain of the mouse with living condition using our TRI system. The *in vivo* measurement area was 3-cm x 1-cm, the total scan time was spent about 4 minutes with 250-μm scanning resolution. And then, the mouse was euthanized after *in vivo* measurement, the whole brain of the mouse was immediately extracted. The total time from anesthetizing to extracting the whole brain was spent 80 minutes.

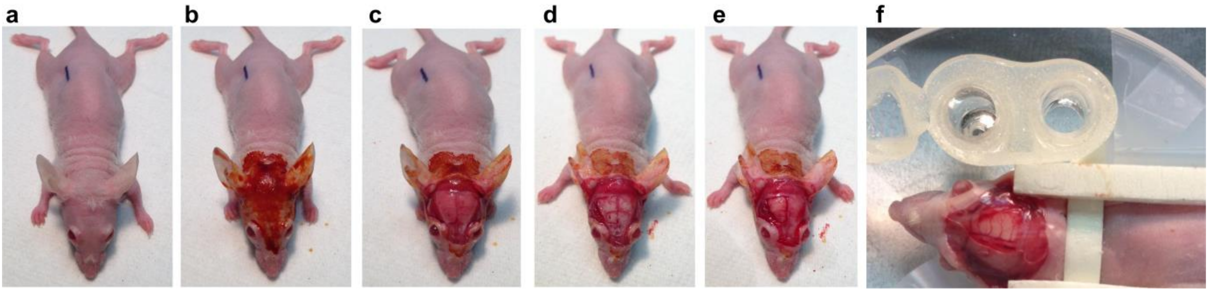

Supplementary Figure 2. Preparations for the in-vivo TRI experiment. (a)-(f) White light images of making a cranial window and keeping contact exposed brain to a quartz window.

3. **Verification of tumor growth from surface to deep brain**

The tumor region in GFP fluorescence image (**Fig. 4g**) seems to be broader than that in THz image (**Fig. 4f**). Because the GFP fluorescence image is an orthogonal projection image, the GFP fluorescence would be visualized not only tumor on the surface but also tumor in the deeper side. We confirmed the broader tumor region on GFP fluorescence image since the tumor was broadly grown from surface to deep brain. The tumor-growth aspect is identified

axial T2-weighted MRI images and H&E images (**Supplementary Fig. 3-4**). We assume the the high- and low-intensity of GFP fluorescence are partially influenced by vertical tumor position. The stronger GFP fluorescence would be detected as the tumor is closer to the surface or denser.

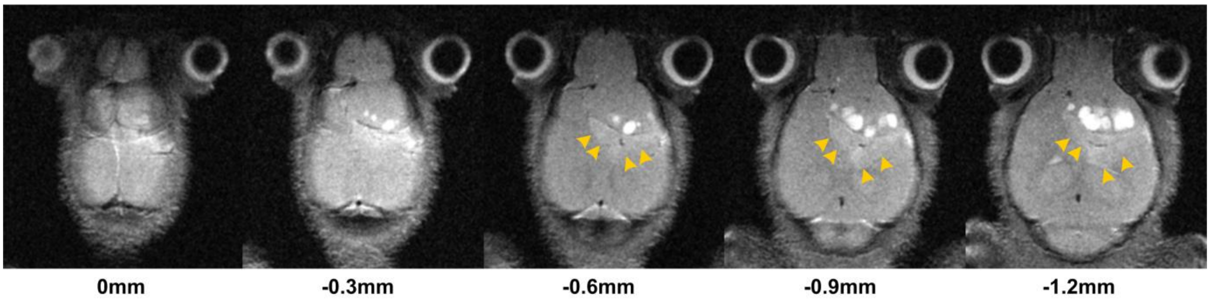

Supplementary Figure 3. T2-weighted axial MRI images. The image obtained from near surface to deeper side with 0.3-mm intervals. The tumor boundary (yellow-orange arrowhead) at -1.2-mm MRI image is broader than the boundary at -0.6mm MRI image.

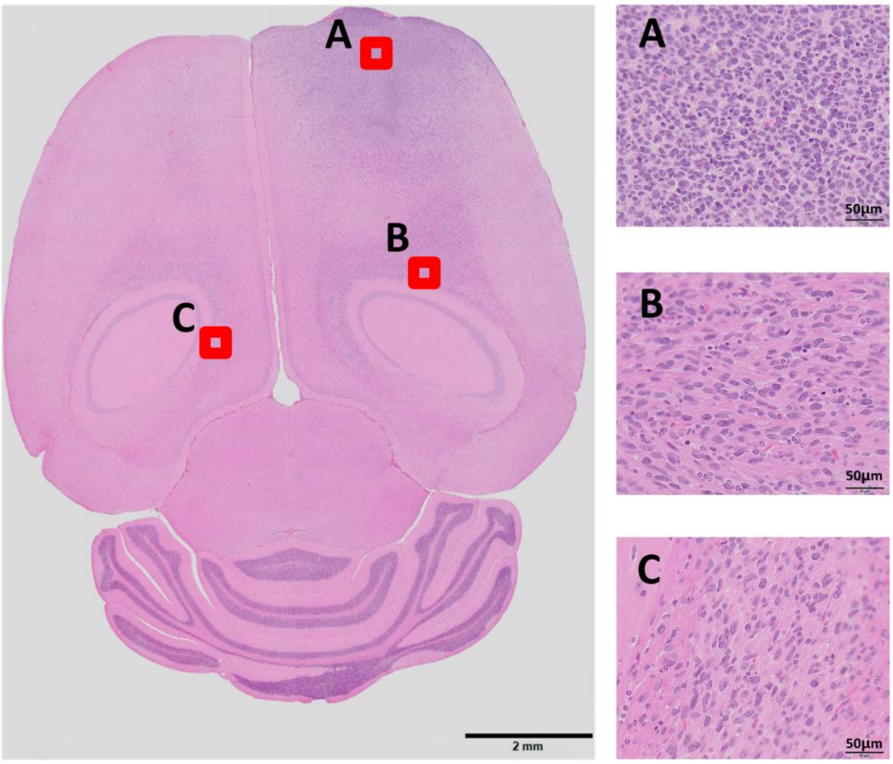

Supplementary Figure 4. H&E image of brain tumor sample which is used in *in vivo* experiment. The image shows an axial image for middle of the brain. The tumor spread to two-thirds of the brain like GFP

fluorescence image (**Fig. 4g**). The tumor density on right-front side of brain (A) is denser than middle sides of brain (B, C). The difference of GFP fluorescence intensity in **Fig. 4g** would be also occurred by tumor cell densities.

4. Schematic diagram of the experimental setup for TRI and detailed description of experiments.

A conventional reflection THz time-domain imaging system with photoconductive antennas was employed in our experiments. We used four off-axis parabolic mirrors to focus THz waves on samples and guide reflected waves to the detector (**Supplementary Fig. 5**). The focal length of used parabolic mirror was 2 inch and the F-number was 1. We didn't use any limiting aperture and the spatial resolution of our system was roughly 0.8-mm at 0.5 THz. The incident angle of THz beams focused through a quartz window was 14.6°. A fast optical delay line with 20 Hz operating frequency and 37 ps time window was used for fast acquisition of THz signals. THz images were obtained with a raster scanning system using a two dimensional (2D) movable sample stage. The acquisition time per image was approximately 26 min and 40 sec for a 50 × 40-mm area with 250-μm scanning resolution. The extracted mouse tumor samples which were cut in two as axial direction and glioma specimens from patients were placed on a quartz sample window, and then we mounted the sample window on a computer-controlled x-y translation stage which was used to raster scan. To measure reference THz signals from water and metal, we made two cylindrical cases using 7-mm diameter plastic pipes and epoxy adhesives as shown in Supplementary Fig. 5. We put the mercury and water into these cases for reference signals in all experiments. The water and metal regions on a THz image were upper side of the image as shown inset of Supplementary Fig. 5. The blue region around the reference region on TRI images shows the epoxy adhesive.

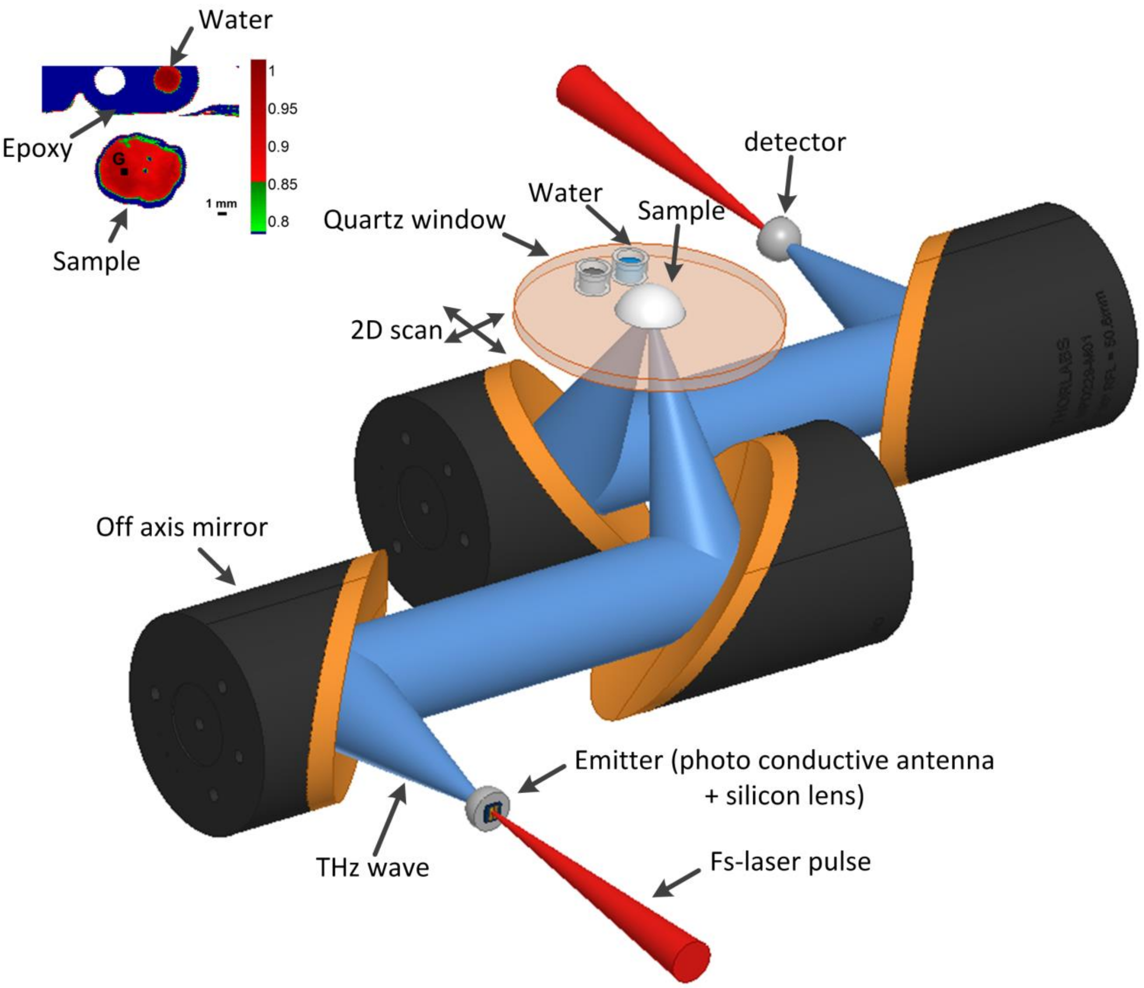

Supplementary Figure 5. Schematic diagram of the experimental setup for TRI. The inset shows a measured example of TRI image which is corresponded to left corner at the bottom of Fig. 2c**.**
